# Generation of Locomotor-Like Activity in the Isolated Rat Spinal Cord Using Intraspinal Electrical Microstimulation Driven by a Digital Neuromorphic CPG

**DOI:** 10.3389/fnins.2016.00067

**Published:** 2016-03-07

**Authors:** Sébastien Joucla, Matthieu Ambroise, Timothée Levi, Thierry Lafon, Philippe Chauvet, Sylvain Saïghi, Yannick Bornat, Noëlle Lewis, Sylvie Renaud, Blaise Yvert

**Affiliations:** ^1^Centre National de la Recherche Scientifique, Institute for Cognitive and Integrative Neuroscience (INCIA), UMR 5287Talence, France; ^2^Institute for Cognitive and Integrative Neuroscience (INCIA), UMR 5287, University of BordeauxTalence, France; ^3^Laboratoire de l'Intégration du Matériau au Système, UMR 5218, University of BordeauxTalence, France; ^4^Centre National de la Recherche Scientifique, Laboratoire de l'Intégration du Matériau au Système, UMR 5218Talence, France; ^5^Bordeaux INP, Laboratoire de l'Intégration du Matériau au Système, UMR 5218Talence, France; ^6^Institut National de la Santé et de la Recherche Médicale, Clinatec-Lab, U1205Grenoble, France; ^7^Université Grenoble Alpes, Clinatec-Lab, U1205Grenoble, France

**Keywords:** hybrid neural networks, neural prostheses, neuromorphic hardware, rehabilitation

## Abstract

Neural prostheses based on electrical microstimulation offer promising perspectives to restore functions following lesions of the central nervous system (CNS). They require the identification of appropriate stimulation sites and the coordination of their activation to achieve the restoration of functional activity. On the long term, a challenging perspective is to control microstimulation by artificial neural networks hybridized to the living tissue. Regarding the use of this strategy to restore locomotor activity in the spinal cord, to date, there has been no proof of principle of such hybrid approach driving intraspinal microstimulation (ISMS). Here, we address a first step toward this goal in the neonatal rat spinal cord isolated *ex vivo*, which can display locomotor-like activity while offering an easy access to intraspinal circuitry. Microelectrode arrays were inserted in the lumbar region to determine appropriate stimulation sites to elicit elementary bursting patterns on bilateral L2/L5 ventral roots. Two intraspinal sites were identified at L1 level, one on each side of the spinal cord laterally from the midline and approximately at a median position dorso-ventrally. An artificial CPG implemented on digital integrated circuit (FPGA) was built to generate alternating activity and was hybridized to the living spinal cord to drive electrical microstimulation on these two identified sites. Using this strategy, sustained left-right and flexor-extensor alternating activity on bilateral L2/L5 ventral roots could be generated in either whole or thoracically transected spinal cords. These results are a first step toward hybrid artificial/biological solutions based on electrical microstimulation for the restoration of lost function in the injured CNS.

## Introduction

Following spinal cord injury, neural prosthesis using electrical stimulation of the spinal circuitry below the lesion can be considered to restore functional locomotor capabilities (Giszter, 2008; Borton et al., 2013; Nishimura et al., 2013). These approaches rely on the ability of intraspinal central pattern generators (CPGs) to generate locomotor rhythms even when isolated from descending inputs, as first assessed *ex vivo* (Brown, 1911; Grillner et al., 1981; Grillner and Wallén, 1985; Cazalets et al., 1992, 1995; Kjaerulff and Kiehn, 1996), and then confirmed *in vivo* in animals and humans (Dimitrijevic et al., 1998; Ichiyama et al., 2005; Minassian et al., 2007). Based on this property, very promising rehabilitation results have been achieved in rodents using epidural stimulation of lumbar segments combined with pharmacological drug applications and rehabilitation training (Courtine et al., 2008, 2009; Musienko et al., 2009; van den Brand et al., 2012). Such approach also allowed a paraplegic patient to achieve assisted standing and stepping movements with full-weight bearing (Harkema et al., 2011).

Because epidural stimulations can activate large networks from the surface of the spinal cord including proprioceptive fibers, a higher degree of control in the activation of the spinal circuitry may be expected using intraspinal microstimulation (ISMS). It has been found in cats that different stereotyped hindlimb mouvements could be elicited by ISMS delivered on single microelectrodes, depending on the position of the microelectrode. Microstimulations delivered in dorsal L5-S1 segments generally elicit hindlimb flexions, while ventral stimulation mostly evokes hindlimb extension (Tai et al., 2003; Lemay and Grill, 2004). More rostral stimulations delivered in the dorsal half of L3-L5 segments also elicit ipsilateral flexion (Barthélemy et al., 2006). Combining ISMS delivered on the dorsal surface of L3-L7 segments with intraveneous injection of the noradrenergic agonist clonidine could further elicit bilateral locomotion (Barthélemy et al., 2006, 2007). Yet, producing sustained locomotion by intraspinal ISMS solely in absence of drugs remains unachieved. Toward this goal, tonic ISMS delivered at 20–50 Hz on microwires implanted in the lumbar ventral horn to target motoneurons of spinalized cats could elicit episodes of hindlimb flexion, extension, and even alternating sequences (Saigal et al., 2004; Guevremont et al., 2006; Lau et al., 2007).

These encouraging results open the way to the design of autonomous neural prosthesis, where coordinated sequences of ISMS control sustained locomotion. For this purpose, artificial neural networks can be used to drive coordinated ISMS sequences, a method recently used to control intramuscular stimulations to restore locomotor behavior (Vogelstein et al., 2008; Mazurek et al., 2012). Here we address this question in the neonatal rat spinal cord isolated *ex-vivo* and interfaced with a penetrating microelectrode array. This preparation can indeed generate locomotor-like activity under pharmacological activation while offering a direct access to intraspinal networks. Here, our goal was to achieve a proof of principle that an artificial neural network can control ISMS to drive locomotor-like activity in this preparation. Two intraspinal sites were identified at L1 level, the alternated stimulation of which generated locomotor-like activity on bilateral L2/L5 ventral roots. An artificial CPG was then implemented on FPGA to control ISMS on these two sites. This hybrid connection could successfully be used to generate lumbar locomotor-like rhythms in a whole and a transected spinal cord.

## Methods

### Ethics statement

All experimental protocols conformed to recommendations of the European Community Council Directive of November 24, 1986 (86/609/EEC) and local French legislation for care and use of laboratory animals. They were approved by the local ethical committee of Bordeaux under recommendation No A5012083.

### Experimental preparation and recording

**Figure 1:**
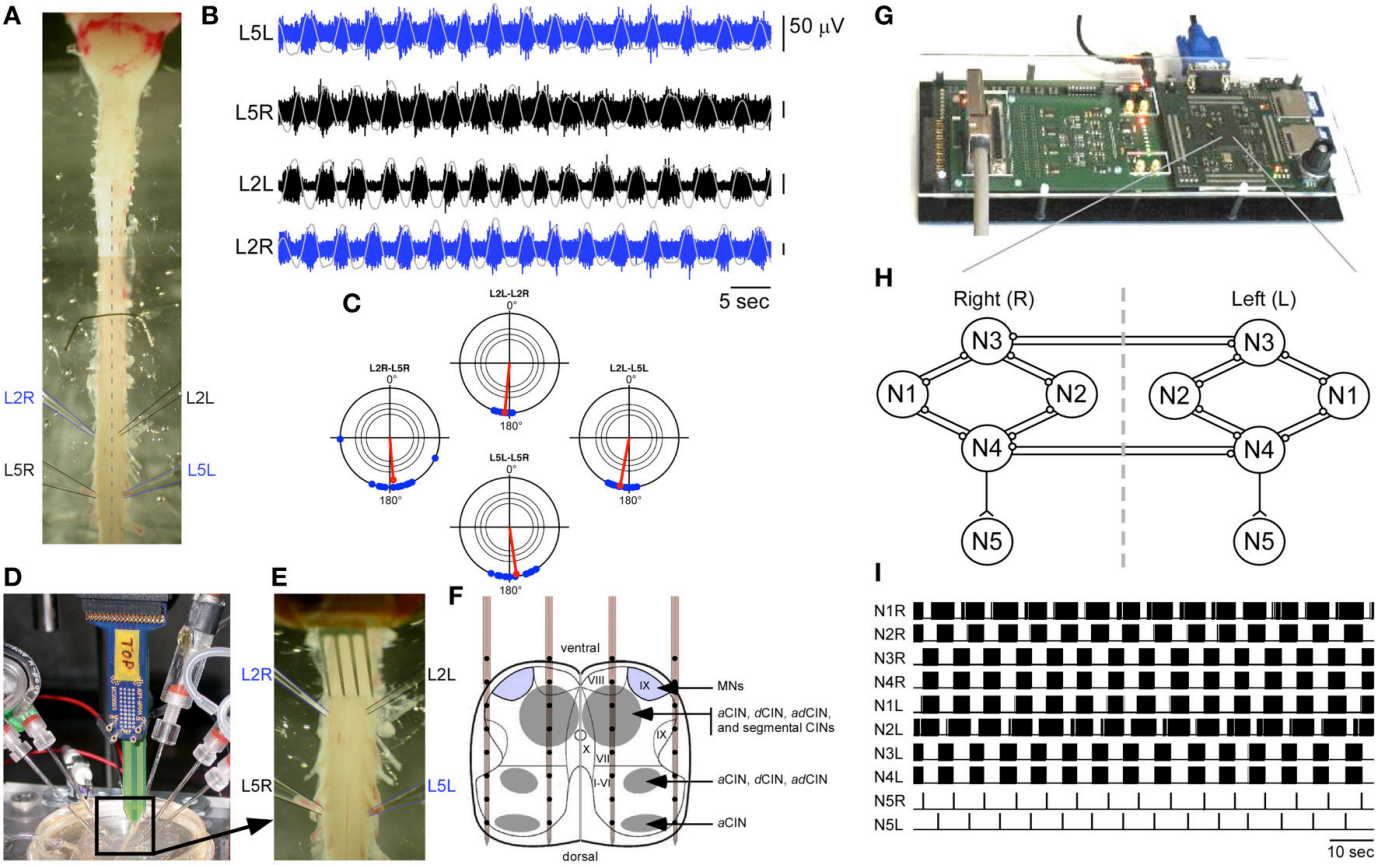
**Experimental paradigm. (A)** Neonatal rat preparation. **(B)** Example of locomotor-like activity on bilateral L2 and L5 ventral roots. Integrated and smoothed traces are superimposed in gray. **(C)** This rhythm can be represented as four polar plots showing the antiphase relationship (180° angle in the plot) between two recordings of same level ipsi and contralateral ventral roots, as well as between two levels on the same side. In each plot, the three concentric circles indicate significance levels of the Rayleigh test at *P* < 0.05, *P* < 0.01, and *P* < 0.001. **(D,E)** View of the experimental setup showing simultaneous ventral root recordings and a 4-shank multichannel neural probe inserted at L1 level for ISMS. **(F)** Schematic representation of the neural probe in the transverse plane of the lumbar spinal cord adapted from Kiehn and Butt (2003). MNs, Motoneurons; CINs, Commissural interneurons; a, ascending; d, descending. **(G)** The multimed platform housing the artificial CPG implemented in an FPGA. **(H)** Structure of the artificial CPG (“o” connections are inhibitory, “<” connections are excitatory). **(I)** Example of the activity of all neurons of the artificial CPG. Neurons N5L and N5R were used to trigger ISMS at the time of every of their spikes.

Whole spinal cord and medulla from newborn Sprague Dawley rats at postnatal stage between P1 and P3 (Figure 1A) were dissected in a cooled artificial CSF (aCSF) solution (pH 7.5) gassed with carbogen (95% O_2_ and 5% CO_2_) and composed of (in mM): 113 NaCl, 4.5 KCl, 2 CaCl_2_2H_2_O, 1 MgCl_2_6H_2_O, 25 NaHCO_3_, 1 NaH_2_PO_4_H_2_O, and 11 D-Glucose. Bilateral L2 and L5 ventral roots were recorded using succion glass electrodes. Ventral root signals were amplified with a gain of 750, band-pass filtered between 0.08 Hz and 3 kHz, and then sampled at a rate of 20 kHz using the previously developed NeuroPXI system (Bonnet et al., 2012), which is an extended version of the former BioMEA system (Charvet et al., 2010). As shown in Figure 1B, this preparation exhibits locomotor-like activity under the application of 5-HT (5 μM), NMA (10 μM), and Dopamine (50 μM), and is thus a good model to explore rehabilitation strategies *in vitro* with an easy access to intraspinal networks. The cords were superfused for 10 min with this cocktail to check that locomotor-like activity could be elicited pharmacology in each preparation prior to hybrid experiments. The drugs were rinsed for at least 60 min to let ventral root activity return to baseline before hybrid experiments started.

### Data processing

Raw ventral root signals were processed as follows to extract spiking activity (see also Heim et al., 2012): for each data sample, a moving average of the signal computed over a 10-ms window centered on this sample was first subtracted from the raw data (DC-removing), and then the obtained value was replaced by the average of the signal computed over a 1-ms time window centered on this sample (smoothing). Signals were then integrated to assess alternating patterns using polar representations and statistics. For this purpose, the signals were first blanked over a period ranging from 15 ms before the stimulation to 150 ms after, and then integrated with a time constant of 0.2 s (see gray traces superimposed on spiking activity in Figure 1B) and finally smoothed with a window of 1 s. The statistical significance of the phase relationship between bursting activity recorded on two different ventral roots was assessed using a Rayleigh test on the delays between the peaks of both integrated signals normalized to 360°. Figure 1C shows the typical polar representation of a locomotor-like activity elicited pharmacologically, with antiphase locking between L2 and L5 ventral roots on each side and left-right L2 or L5 pairs.

### Electrical stimulation

As shown in Figures 1D–F, electrical microstimulations were delivered on single microelectrodes of Neuronexus neural probes made of four shanks separated by 400 μm, each containing eight 30-μm-diameter microelectrodes separated by 100 μm (probe model A4x8-5 mm-100-400-703-A32). Based on earlier studies showing the localization of rat CPGs (Cazalets et al., 1995; Antri et al., 2011), the neural probe was inserted at the L1 level of the spinal cord. Depending on the preparation, between 15 and 26 sites of the probe were individually tested for stimulation. Each stimulation consisted of a train of 10 biphasic pulses separated by 1 ms. Each pulse was charge-balanced with an initial cathodic phase of 500 μs and an amplitude of typically 150–300 μA, immediately followed by a 10 times longer anodic phase of 10 times smaller amplitude. Stimulations were monopolar with respect to a distant electrode in the bath.

### Artificial CPG

An artificial CPG neural network inspired from Hill et al. (2001) was implemented onto a configurable digital integrated circuit (FPGA), supported by an electronic platform designed at IMS (Figure 1G). The implementation of this network is fully described in Ambroise et al. (2013). In brief, the core CPG network shown in Figure 1H consisted of two symmetric sub-networks of four regular spiking neurons (N1L-N4L and N1R-N4R in Figure 1H). These eight neurons have identical biomimetic dynamics following the Izhikevich model (Izhikevich, 2003) and are interconnected by reciprocal inhibitory GABA_*A*_-like synapses. Synaptic efficiency was governed by activity-dependent depression (Tabak et al., 2000). The dynamics of each neuron was thus governed by four parameters a, b, c, and d according to the following differential equations:

$$\begin{array}{l} \frac{dv}{dt}=\frac{v^{2}}{32}+4v+109.375-u+I_{bias}+\overset{Nsynapses}{\underset{i=1}{\sum }}I_{syn}^{i} \end{array}$$

$$\begin{array}{l} \frac{du}{dt}=a\left(bv-u\right) \end{array}$$

with the after-spike resetting condition:

fv≥30mV then{v←cu←u+d

where the other parameter *I*_*bias*_ is a constant bias current and *I*^*isyn*^ is the synaptic current received from the ith input neurons. For each input, the synaptic current *I*_*syn*_ is governed by the following equation:

$$\begin{array}{l} \frac{dI_{syn}}{dt}=-\frac{I_{syn}}{\tau_{syn}}+\left(1-\lambda_{syn}\right)\cdot W_{syn}\cdot \delta \left(t-t_{spike}\right), \end{array}$$

where τ_syn_ is the synaptic current decay time constant, δ(*t* − *t*_*spike*_) = 1 if the input neuron produces a spike at time *t* and 0 otherwise, and λ_*syn*_ is the synaptic efficiency. The synaptic efficiency was dynamic with a decay time constant τ_*reg*_ and synaptic depression modeled through the following equations:

$$\begin{array}{l} \frac{d\lambda_{syn}}{dt}=-\frac{\lambda_{syn}}{\tau_{reg}}\cdot \left(1-\delta \left(t-t_{spike}\right)\right)+P\cdot \left(1-\lambda_{syn}\right)\delta \left(t-t_{spike}\right), \end{array}$$

where *P* is a dissipation percentage parameter decreasing synaptic efficiency after each spike.

The design was optimized to cost few digital resources on the FPGA while running in real-time at the ms resolution (Ambroise et al., 2013). Both sub-networks of the CPG produced alternating rhythmic bursting activity (Figure 1I). The resulting bursting activity was integrated for each sub-network by a fifth spiking neuron (N5L and N5R in Figure 1H), producing only one spike per burst, and representing the CPG output neurons. In the current system version, each parameter change in the neural network model requires a new synthesis of the FPGA configuration, which is done by the implementation of a new configuration file (bit file) from the computer to the FPGA, a procedure taking several seconds. Further versions will allow dynamic reconfiguration of the FPGA, thus allowing online synaptic adaptation for example.

### Hybridization of the artificial CPG to the spinal cord

The CPG output neurons produced a rhythmic left-right alternating activity made of 1 spike per cycle on each network side. These output spikes were used to trigger intraspinal electrical microstimulations on two different microelectrodes of the neural probe, one on each side of the spinal cord.

### Statistical analysis

The statistical significance of alternating patterns of bursting activity recorded on two ventral roots VR1 and VR2 was assessed by a circular Rayleigh test performed on the angular values of individual bursts. The amplitude of the Rayleigh statistics was compared to three significance levels (*P* < 0.05, *P* < 0.01, and *P* < 0.001) represented as different circles on the polar representations in Figures 1C, **3C**, **4C**. The angular values on which the test was performed were computed as follows. The peaks of integrated signals were first detected on each ventral root for each burst. Then, for each peak of VR2 occurring at time t2, we considered the two neighboring peaks occurring before and after t2 on VR1 (t1_prev ≤ t2 and t1_after ≥ t2) to compute a local angular value using the following relation:

$$\begin{array}{l} \phi =\frac{\pi }{2}+2\pi \frac{t_{2}-t_{1prev}}{t_{1after}-t_{1prev}} \end{array}$$

## Results

### Identification of two intraspinal microstimulation sites

**Figure 2:**
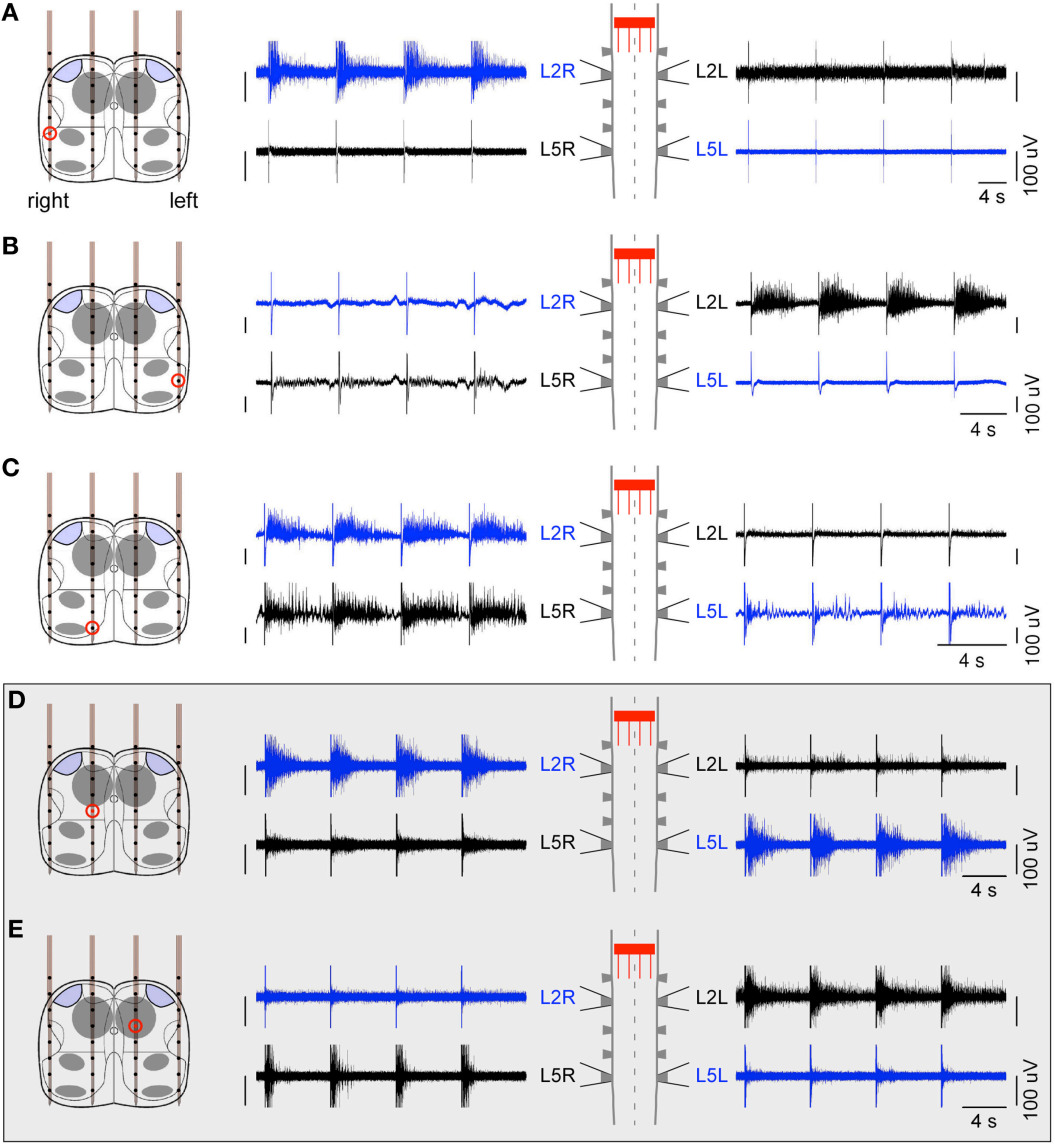
**Ventral root responses to ISMS at L1 level**. ISMS elicited different responses depending on the site of stimulation. **(A,B)** Dorsolateral stimulations elicited a burst response mainly on the ipsilateral L2 ventral root. **(C)** Example of a more medial stimulation eliciting a response on ipsilateral L2 and L5 ventral roots. **(D,E)** Importantly, ISMS delivered medially on either side of the central canal elicited a burst response on the diagonal L2 ipsilateral and L5 contralateral. These specific intraspinal locations were found consistently across preparations and thus further used for the hybrid experiments. For all panels, a sequence of four stimulations is illustrated.

An initial set of 10 preparations were used to identify the best levels of the spinal cord to target between T11 and L5 in order to elicit consistent responses on the L2/L5 ventral roots. This was done using either a single glass pipette microelectrode or a penetrating shank. We found that ISMS delivered at the L1 level were the most reliable. We then considered 6 other preparations in which we inserted a 4 × 8 probe transversally at the L1 level. Between 15 and 26 contacts were scanned successfully at different current intensity levels between 40 and 800 μA. We initially tested classical symmetrical biphasic pulses (cathodic-first with a cathodic phase of 500 μs), and found that they were less efficient than balanced but non-symmetrical pulses where the anodic phase was twice weaker and longer. Cathodic current intensities above 150 μA were generally required to obtain reliable responses on the ventral roots. As shown in Figure 2, we found that ISMS at L1 level elicited different burst responses on bilateral L2/L5 ventral roots depending on the location of the stimulation site. ISMS delivered dorsolaterally elicited a burst on the ipsilateral L2 ventral root (Figures 2A,B). Stimulations delivered dorsomedially typically elicited a response on both ipsilateral L2 and L5 ventral roots (as in Figure 2C), and occasionally also on the contralateral L5 ventral root. Consistently across preparations, we identified two intraspinal sites, one on each side of the spinal cord located about 200 μm laterally from the midline and approximately at a median position dorso-ventrally near the central canal (about 400 μm from the dorsal surface). We found that a stimulation delivered on either site elicited a burst response simultaneously on the ipsilateral L2 and contralateral L5 motor outputs (Figures 2D,E). This result thus opened the possibility to generate locomotor-like activity using coordinated stimulation between these two stimulation sites.

### Generation of alternating patterns using the artificial CPG

The eight neurons of the core network had the following identical parameters that were found to lead to a robust alternating activity on the output neurons of the CPG: *a* = 0.02, *b* = 0.2, *c* = −65, *d* = 8, and *I*_*bias*_ = 8. The time constant τ_*syn*_ of the exponential decay of synaptic current was set to 100 ms. For output synapses from neurons N2R and N2L, we used *W*_*syn*_ = −1.26 and *P* = 0.1%; for input synapses onto neurons N2R and N2L, we used *W*_*syn*_ = −3 and *P* = 0.03%. For all other inhibitory synapses, we used *W*_*syn*_ = −3.8 and *P* = 0.03%. The output layer neurons N5R and N5L received excitatory AMPA-like connections respectively from N4R and N4L, both defined by *W*_*syn*_ = 30 and *P* = 90%. The initial conditions were *v* = −20 and *u* = −4 for N1R, N3L, N4L, N2R neurons, *v* = 0 and *u* = 0 for N1L, N3R, N4R, N2L, and *v* = −65 and *u* = −13 for N5R and N5L. With these parameters, each CPG bursting activity pattern generated one spike on N5L and one spike on N5R, used as trigger for delivering microstimulation to the spinal cord. The stimulation is only triggered by the CPG output and consisted of a short train of 10 stimuli (see Methods). By changing τ_*reg*_ (from 4 to 12 s), the artificial CPG alternating period could be modulated between 2 and 7 s.

### Generation of locomotor-like activity in a whole *ex-vivo* spinal cord using a hybrid connection

**Figure 3:**
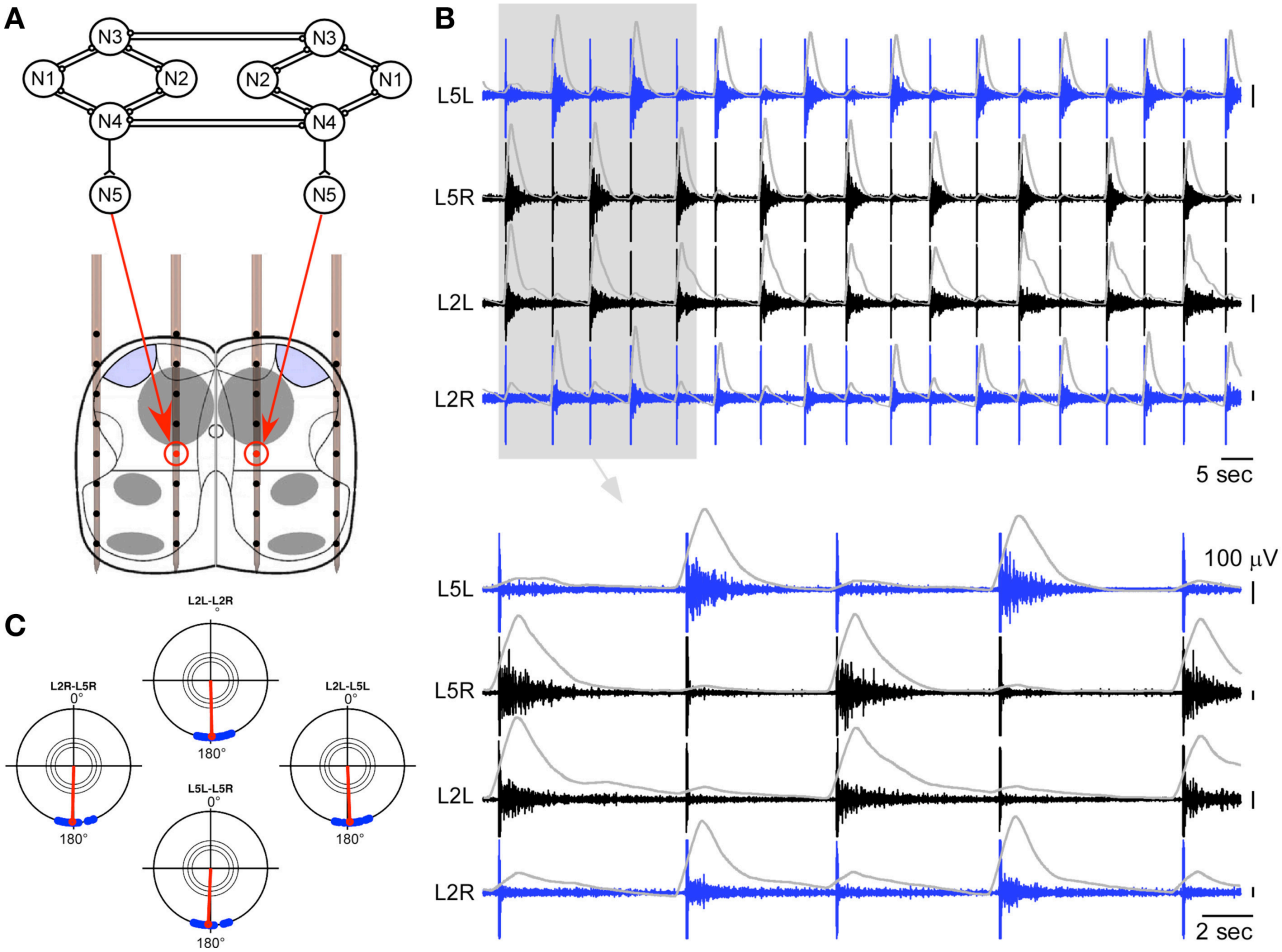
**Hybrid experiment in the case of a whole spinal cord. (A)** Schematic representation of the hybrid connection between the output neurons of the artificial CPG and the two intraspinal stimulation sites. **(B)** Ventral root responses to a sequence of ISMS (150 and 200 μA for the cathodic phase on the left and right sites, respectively) driven by the artificial CPG at a cycle frequency of 6.7 s (integrated traces shown in gray), showing clear left-right and ipsilateral alternating patterns (top: 2 min, bottom 30 s). **(C)** Polar representation of this induced rhythm as in Figure 1C.

In a first step, we made a hybrid connection between the artificial CPG and a whole spinal cord. The output neuron of each side of the artificial CPG controlled intraspinal stimulations on one of the intraspinal stimulation site (Figure 3A). Each spike of an artificial output neuron triggered one stimulation on the corresponding intraspinal site. Using this strategy, clear locomotor-like activity could be obtained on bilateral L2 and L5 ventral roots (Figures 3B,C). Once the artificial CPG was turned on, this activity established at the first or second stimulation, remained robust with a 1:1 correspondence to the artificially imposed pace as long as the artificial CPG was ON, and then vanished as soon as the CPG was turned off.

### Generation of locomotor-like activity in a transected spinal cord *ex-vivo* using a hybrid connection

**Figure 4:**
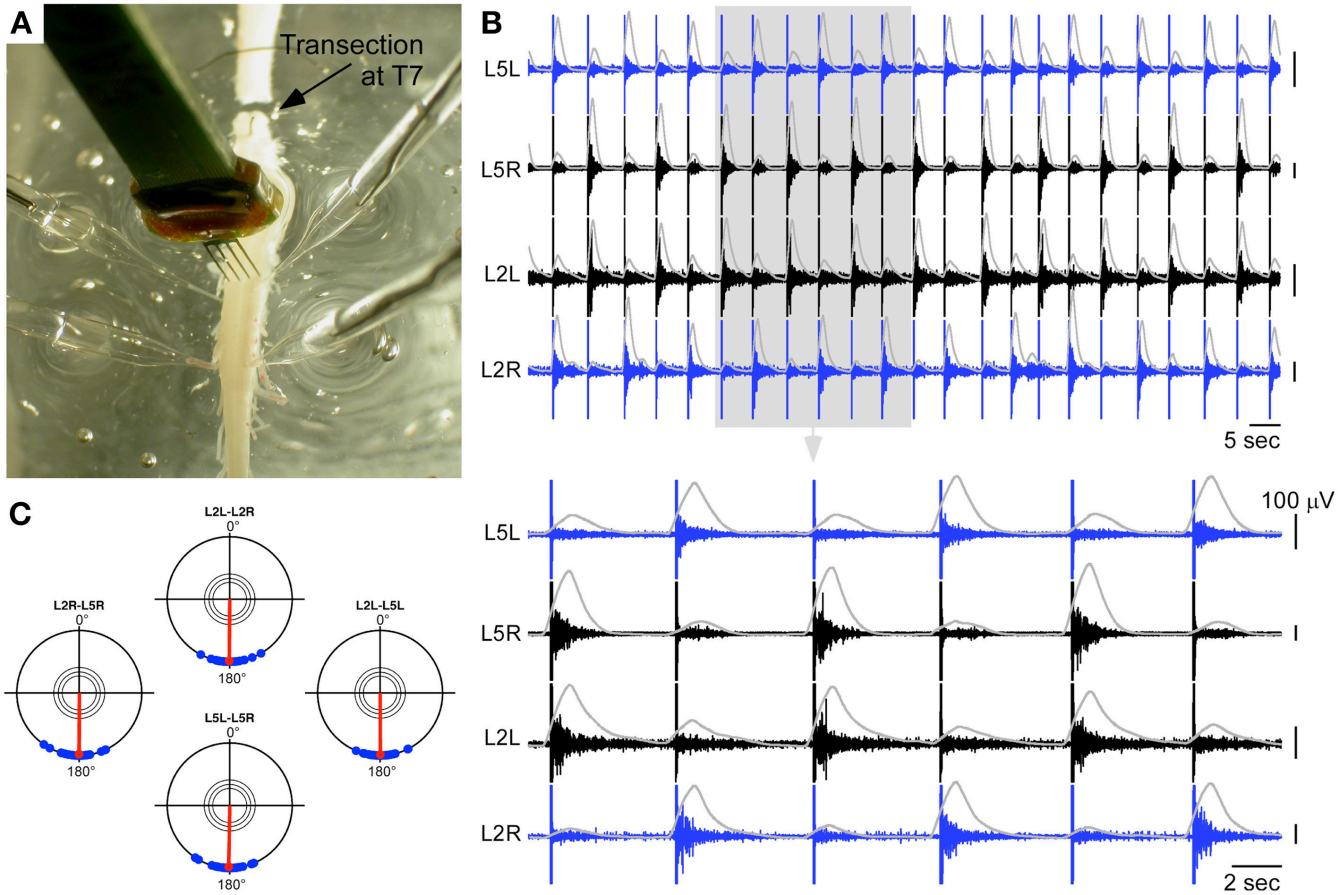
**Hybrid experiment in the case of a spinal cord transected at T7. (A)** Picture of the experiments showing the transection, the 4 ventral root recordings, and the neural probe used to deliver ISMS. **(B)** Ventral root responses to a sequence of ISMS (300 μA for the cathodic phase on each site) driven by the artificial CPG at a cycle frequency of 5.2 s (integrated traces shown in gray), showing clear left-right and ipsilateral alternating patterns (top: 2 min, bottom 30 s). **(C)** Polar representation of this induced rhythm as in Figure 1C.

In a second step, we tested whether this result could also be obtained in a lumbar spinal cord fully isolated from descending inputs to mimic a lesion situation. We thus considered another preparation, which was transected at the T7 level (Figure 4A). The same type of hybrid connection was used between the artificial CPG and the intraspinal stimulation sites. As shown in Figures 4B,C, this strategy could also successfully elicit locomotor-like activity on bilateral L2 and L5 ventral roots. The locomotor pattern could be maintained for more than 7 min as long as the artificial CPG was ON. Moreover, several periods of alternating cycle were tested in this preparation spanning the range allowed by the artificial CPG (2.6, 3.1, 4, 5.2, and 6.7 s between successive left and right stimulations). We found that the spinal cord could follow the imposed rhythm in an exact 1:1 correspondence at these different frequencies for the whole durations of the hybrid connection tested: 560 s at of 6.7 s (84 left+right stimulations), 430 s at 5.2 s (85 stimulations), 369 s at 4 s (97 stimulations), 392 s at 3.1 s (123 stimulations), and 363 s at 2.6 s (217 stimulations). The artificial CPG was limited to these period values and we thus could not test higher speeds to see when the spinal cord would stop following the artificial network. Nevertheless, the tested frequencies covered the pace of a pharmacologically-evoked rhythm with inter-burst period around 5 s on each ventral root (as shown in Figure 1B).

## Discussion

The goal of this study was to test whether ISMS controlled by an artificial neural network could in principle be used to restore locomotor-like activity in a transected spinal cord. This proof of principle was achieved here in the neonatal rat spinal cord isolated *ex vivo*, using the advantage that this preparation can display locomotor rhythms while offering a direct access to intraspinal networks. This paradigm allowed identifying appropriate intraspinal sites for stimulation, which were localized on either side of the central canal at L1 level. Efficient stimulations could be obtained for current levels of the order of 150–300 μA. These current amplitudes were higher than those generally used in previous *in vivo* studies to elicit limb movements using ISMS in cats (Guevremont et al., 2006) and rats (Shahdoost et al., 2014). One possibility could be the difference in the frequency of the pulse train between these studies and our paradigm. Here we used only 1 ms between two successive pulses of the same train, while *in vivo* ISMS typically used lower frequency 40–50-Hz trains, likely to be more efficient. Moreover, the level of excitability of the CPGs might be different between our *ex vivo* situation at room temperature and the *in vivo* context where the complete network including sensory feedbacks remain present. Also, the activity elicited here on the ventral roots did not result from a direct activation of the motoneurons since a stimulation delivered on a precise site in L1 elicited activity simultaneously on the ipsilateral L2 and the contralateral L5. Thus, we created an indirect activation of the motoneurons, likely by activating a group of interneurons participating in the lumbar CPG. It is possible that higher stimulation intensities are required to robustly activate the whole CPG rather than generating more discrete muscle contractions. As a result and based on previous studies (Ranck, 1975; Yeomans et al., 1986; Joucla et al., 2012), it is likely that the currents we used typically activated neurons within a distance of about 250–500 μm from the electrode. Nevertheless, we could verify experimentally the specificity of our ISMS. Indeed, many electrode positions of the same probe were tested for stimulation and, as illustrated in Figure 2, the response to ISMS on the four ventral roots greatly depended on the site of stimulation. In particular, there was a clear dissociation of the response for the two stimulation sites that we identified on each side of the central canal, which were separated by typically only 400 μm and yet triggered completely different output patterns (opposite L2-L5 diagonals). Using adjacent sites of the same shanks distant by only 100 μm from the optimal stimulation sites did not produce consistent activation of the L2-L5 diagonal. Actually, the fact that we were able to reproduce locomotor-like activity patterns by coordinating ISMS on these two sites was precisely based on this dissociation.

The localization of the two intraspinal sites was in the region of the lumbar CPGs, which have previously been localized at the upper lumbar region within T13 and L2 segments (Cazalets et al., 1995; Kjaerulff and Kiehn, 1996). The ISMS positions identified here fall in this region and are close to several classes of projecting commissural interneurons (Kiehn and Butt, 2003). Moreover, these locations are also consistent with a high density of neurons involved in alternating locomotor activity, identified between L1 and L2 levels with calcium imaging (Antri et al., 2011). Altogether this suggests that ISMS may activate elements of the lumbar CPGs responsible for the coordination of opposite L2 and L5 motoneuronal pools, maybe through the direct activation of commissural interneurons. It should be noted that each stimulation typically triggered only one burst episode on output roots (as clearly seen in Figures 2–4). Thus, all bursts were followers of the stimulation pulses. In particular, no wind-up was observed since bursts disappeared as soon as the stimulation stopped, and the frequency of the locomotor pattern could be imposed by the frequency of the artificial CPG. For these reasons, it is possible that each stimulation activated a group of interneurons participating in the lumbar CPG, and that the coordination of two stimulation sites could allow reproducing locomotor-like activity by the coordinated activation of these groups.

The quest for fully autonomous neural prosthesis based on hybrid connections between the CNS and artificial neural networks is a tremendous challenge. Here, we only performed a first step toward this goal by making a unidirectional (open-loop) connection between an artificial CPG and the spinal cord circuitry. In this work, the artificial network was initially configured with proper parameters to exhibit adequate alternating rhythmic activity. In particular, modifying the frequency of the rhythm required a manual reconfiguration and re-synthesis of the network in the FPGA. Further versions of the CPG hardware implementation will offer the possibility to modulate its dynamics in real time. This will then open the possibility to build bidirectional hybrid connections, where supralesional activity can be used to control the artificial network dynamically in order to achieve a close loop artificial connection over the lesion. In particular, future developments could include paradigms where the artificial network would be dynamically controlled and/or modulated by inputs from supraspinal and/or intraspinal supralesional information, and possibly modulated in real time by sensory feedbacks produced after ISMS in preparations preserving whole hindlimbs attached to the spinal cord.

As a further perspective, such hybrid approach will also need to be extended *in vivo* in order to assess whether it can also help recover locomotion capabilities in adult animals subject to chronic spinal cord lesions. While this has started to be addressed using ISMS in spinal cats (Guevremont et al., 2006; Mazurek et al., 2012), little ISMS work has yet been obtained in rats (Shahdoost et al., 2014). Previous lesion results in adult rats showed that destruction of the gray matter at T13-L2 level induce severe locomotor deficits, while more caudal lesions have more limited influence (Magnuson et al., 2005), suggesting a localization of neonatal lumbar CPGs consistent with those of adult animals. Yet, whether the two stimulation sites identified in this study remain conserved in adulthood to elicit locomotor movements in spinal animals remain to be tested. Moreover, in the case of chronic animal models of paralysis, lesions may induce remodeling of intraspinal circuits on the long term (Dietz and Müller, 2004), which may change the way networks could be activated. However, previous studies indicate that despite such plastic changes, lumbar CPG circuitry remain present and can be reactivated below the lesion with training to recover functional locomotion (Barrière et al., 2008; van den Brand et al., 2012). An open question is whether such rehabilitation perspective can also be obtained using active neural prosthesis solely based on ISMS. Chronical experiments will thus be necessary to determine if the spinal circuitry below the lesion may also be exploited through hybrid connections with artificial neural networks to recover locomotor functions with autonomous spinal neural prosthesis.

## Conclusion

In conclusion, the present study is a first demonstration of a hybrid interconnexion between a living spinal cord and an artificial neural network driving ISMS to restore functional activity. These results are a first step toward intelligent neural prostheses based on hybrid live/artificial connections for the restoration of lost function in the injured CNS.

## Author contributions

SJ, SR, and BY designed the study. MA, TLe, SS, YB, NL, and SR designed the artificial CPG and associated electronics (Multimed system). SJ, MA, TLa, PC, and BY developed the experimental setup. SJ and MA performed the experiments. SJ and BY analyzed the data. SJ, TL, SS, NL, SR, and BY wrote the manuscript.

### Conflict of interest statement

The authors declare that the research was conducted in the absence of any commercial or financial relationships that could be construed as a potential conflict of interest.
